# Cecum perforation by plug migration: an unexpected late complication of inguinal hernia mesh repair

**DOI:** 10.1093/jscr/rjad137

**Published:** 2023-03-14

**Authors:** Filipa Policarpo, Ana Alves Rafael, Miguel Fróis Borges, Fernando Azevedo

**Affiliations:** Department of General Surgery, Centro Hospitalar Lisboa Ocidental, Lisboa, Portugal; Department of General Surgery, Centro Hospitalar Lisboa Ocidental, Lisboa, Portugal; Department of General Surgery, Centro Hospitalar Lisboa Ocidental, Lisboa, Portugal; Department of General Surgery, Centro Hospitalar Lisboa Ocidental, Lisboa, Portugal

## Abstract

Case of a 79-year-old male previously submitted to Rutkow-Robbins inguinal hernia repair. He presented himself at the Emergency Room with an inguinal inflammatory mass and bowel obstruction for 5 days. A strangulated recurrent inguinal hernia was assumed and emergency surgery was performed. Since an inguinal abscess was present, a midline laparotomy was performed. The previous polypropylene plug was found in an intraperitoneal position, fistulizing to the cecum and creating a 2 cm wide perforation, without intraperitoneal collections or bowel compromise. An *en bloc* atypical resection of the cecum with the plug was performed and the abdominal wall abscess was drained. The patient had a slow, but uneventful postoperative course. Given the rarity of cases, the high variability of clinical presentation and the potential seriousness of mesh migration complications, the authors review the topic of mesh migration.

## INTRODUCTION

One of the landmarks in Abdominal Wall Surgery was the development of meshes in treatment. However, their use is not free of risks and can lead to serious complications [1, 2]. One of these risks is mesh migration [1].

With a range of clinical presentations as wide as the anatomic locations that the mesh can acquire, 91% of the patients who present with this condition need a surgical intervention [1] with eventual organ ressection [3].

Considering the rarity and the potential seriousness of this complication, a larger database of reported clinical cases seems necessary to develop a more detailed study and guidelines regarding the approach to these patients.

Inspired by a particular clinical case of cecum perforation by plug displacement, the authors review migration of abdominal wall meshes.

## CASE REPORT

We present the case of a 79-year-old male, with a known history of atrial fibrillation, chronic obstructive pulmonary disease due to smoking (46 pack-years), submitted to a bilateral hernia mesh repair in 2015: Lichtenstein technique on the left side and Rutkow-Robbins technique on the right side, the latter due to an European Hernia Society PL3 inguinal hernia.

The patient presented himself at the Emergency Department in 2018 with a 1-week painful right inguinal mass with inflammatory signs, located 3 cm above the inguinotomy scar and associated with bowel obstruction for the past 5 days. He was previously medicated with flucloxacillin without improvement.

There being no other symptoms or signs, an abdominal wall abscess secondary to a strangulated recurrent inguinal hernia was assumed.

Blood tests showed leukocytosis (13 100 x 10^6^/L), neutrophilia (80%) and an elevated C-reactive protein value (20.6 mg/dL).

**Figure 1:**
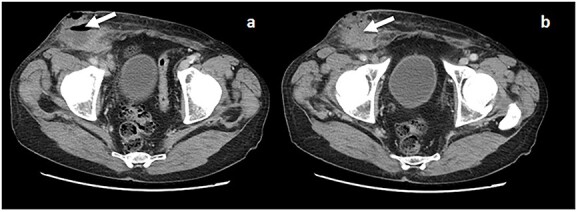
(**a** and **b**) CT scan with evidence of a fluid collection in the right inguinal area (arrow) and an apparent recurrent hernia.

Computerized tomography (CT-scan) revealed an apparent right recurrent inguinal hernia with satellite regional lymph nodes of 15 mm and an 8-cm fluid collection and air in the subcutaneous tissue (Fig. 1a and b).

**Figure 2:**
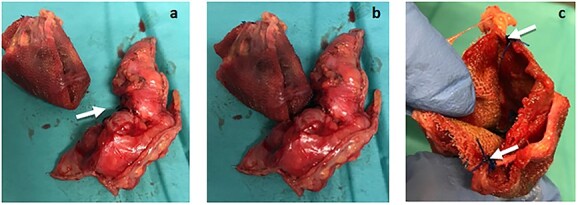
(**a**) The plug removed from the peritoneal cavity (left) and the cecum atypical resection specimen (right), where the 2 cm perforation area can be seen (arrows). (**b**) *Plug* and cecum positioned according to its intra-operatory location, in which the erosion of the cecum wall was observed in direct contact with the plug*.* (**c**) *Plug* still with two fixation points (arrows).

An exploratory midline laparotomy was performed. It revealed a 2 cm cecum perforation caused by direct contact with the polypropylene plug (Fig. 2a–c), which had an intra-peritoneal position at the entrance of the internal inguinal orifice—still with two fixation sutures (previously described as polypropylene). No intraperitoneal collections or bowel compromise were observed.

We performed an atypical resection of the cecum *en bloc* with the plug. The abdominal wall abscess was drained through a right inguinotomy. It was not possible to remove the flat portion of the mesh due to its strong incorporation in the abdominal wall.

The pathological analysis of the cecum atypical resection specimen documented ischemic focal alterations with erosions, congestion, inflammatory infiltrate and granulation tissue.

The microbiological exam of the purulent exudate identified intestinal flora microorganisms.

The postoperative period was noteworthy for a surgical site infection, treated with ampicillin, metronidazole and fluconazole directed against the isolated agents (*Enterococcus faecium*, *Enterococcus avium*, *Escherichia coli*, *Bacteroides fragilis* and *Candida albicans*) and a negative pressure therapy device, enabling the wound to be completely closed afterwards.

The patient was discharged 25 days postoperatively.

## DISCUSSION

Mesh migration is a rare complication of hernia surgery. It is usually defined as mesh location in an anatomic area different from the intended one [1]. This complication can occur with any mesh, regardless of mesh material, hernioplasty technique or fixation suture [1, 3].

The prevalence of mesh migration is unknown. This can be explained by the fact that the majority of the evidence related to this situation is based on case reports and a paucity of data [1].

**Figure 3:**
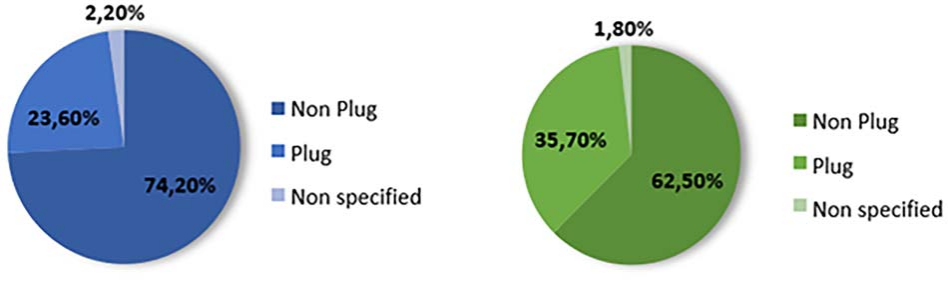
Distribution of *mesh plugs* regarding the migration of hernia meshes in general (left) and specifically in inguinal hernia meshes (right) [1].

Nonetheless, a review of published reports by the authors concluded that the majority of cases correspond to male patients (76%), on average 59.8 years old, and having been submitted to inguinal hernia surgery (62.9%) [1] (Table 1). It was also noted that plug migration corresponds to 23% of the published cases related to abdominal wall mesh migration in general and 35% of the inguinal mesh migration in particular [1] (Fig. 3).

**Table 1:** Distribution of the mesh migration report cases according to the *index* surgery [1]

Distribution of mesh migration cases	
Hernia location	Published cases
Inguinal	62.9%
Incisional	28.1%
Umbilical	6.7%
Others	2.2%

There is a wide range of clinical presentations since the movement of the mesh allows direct contact with different organs [1], which may manifest as bowel obstruction, intra-abdominal adhesions, recurrent urinary infections, hematuria, enteric or enterovesical fistulas or abdominal/pelvic collections [3].

**Figure 4:**
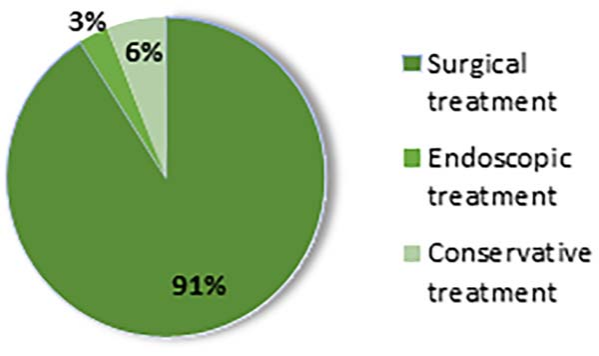
Therapeutic approach of the published cases of mesh migration [1].

It is estimated that 91% of mesh migration cases need new surgical intervention to treat the related symptoms [1] (Fig. 4), which on average takes place 4 years after the hernia surgery, there being described cases with time intervals of between 2 and 7 years [1].

These postoperative complications can be resolved with a simple mesh removal or *in extremis* may require organ resection [3].

Some authors classify mesh migration as primary or secondary [1, 3].

Primary migration occurs due to essentially mechanical causes, and is the movement of the mesh through the weak points of the surrounding tissues, generally due to inappropriate fixation or to external forces that cause the movement of the mesh [1, 3].

Secondary migration occurs due to foreign body reaction and subsequent erosion of the nearby structures, allowing slow and gradual mesh displacement through the different anatomic planes [1, 3]. This process is mostly related to the mesh material and the type of fixation used, being more frequent with polypropylene meshes. However, some authors argue that this association is because these meshes are the type most used.

A mixed mechanism is also found in which, after a primary migration, the erosion of tissues was not initially in contact with the mesh [3].

In the case described, considering the intra-operatory visualization of two non-absorbable (*Prolene*) fixation points of the mesh, the origin of the mesh migration was interpreted as a foreign body reaction. This reaction induced erosion of the posterior wall of the inguinal canal, enabling plug migration to the peritoneal cavity and subsequent cecum adhesion and perforation.

## CONFLICT OF INTEREST STATEMENT

None declared.

## FUNDING

None.

## DATA AVAILABILITY

All the data are presented in the main manuscript or additional supporting files.
